# Comparing two types of platelet-rich fibrin membranes in the management of dehiscence defects with simultaneous implant placement in the posterior mandible: a randomized controlled trial

**DOI:** 10.1186/s12903-026-08043-w

**Published:** 2026-03-27

**Authors:** Mohamad Mhdy A. Abla, Ahmed M. Shaaban, Passent A. Younis

**Affiliations:** Department of Oral and Maxillofacial Surgery, Faculty of Dentistry, Alexandria University, Alexandria, Egypt

**Keywords:** Platelet-rich fibrin, Extended PRF, Xenograft, Guided bone regeneration, Implant

## Abstract

**Background:**

Alveolar ridge resorption after tooth extraction may compromise implant placement and frequently necessitates guided bone regeneration (GBR). Platelet-rich fibrin (PRF) is widely used in oral surgery; extended PRF (e-PRF) has been proposed to prolong growth factor availability and membrane stability. This randomized clinical trial compared PRF and e-PRF membranes combined with bovine xenograft for horizontal ridge augmentation with simultaneous implant placement in the posterior mandible.

**Methods:**

Twenty patients with posterior mandibular ridge deficiencies (residual ridge width 4–6 mm) were randomized (1:1) to receive xenograft + PRF (control, *n* = 10) or xenograft + e-PRF (study, *n* = 10) at the time of implant placement. Primary outcome: horizontal bone width gains at 6 months assessed by standardized CBCT measurements 1 mm apical to implant platform. Secondary outcomes: implant stability quotient (ISQ) at baseline and 6 months, postoperative pain (VAS), and edema. Sample size was calculated to detect the expected difference in ISQ change with 80% power and α = 0.05. Statistical tests included paired and independent t-tests, repeated measures analyses, and non-parametric tests where appropriate. Significance threshold was *p* ≤ 0.05.

**Results:**

Twenty patients (30 implants; 8 males, 12 females; mean age 40.2 ± 5.3 years) completed the study. Baseline characteristics and residual ridge widths were similar between groups. Implant stability (ISQ) increased significantly within both groups from baseline to 6 months (*p* < 0.001) but did not differ between groups at either time point (baseline *p* = 0.622; 6 months *p* = 0.263). Postoperative pain and edema followed similar, transient patterns in both groups (no significant between-group differences). At 6 months, horizontal bone gain was significantly greater in the e-PRF group (mean gain 2.28 ± 0.79 mm) than in the PRF group (0.75 ± 0.18 mm), *p* < 0.001.

**Conclusions:**

In this randomized clinical trial, both PRF and e-PRF combined with bovine xenograft and simultaneous implant placement produced favorable clinical outcomes, while e-PRF provided superior horizontal bone gain at 6 months. Further larger and longer-term trials with prospective registration are recommended to confirm these findings.

**Trial registration:**

The study protocol approved by the Institutional Review Board of the Faculty of Dentistry, Alexandria University, Egypt (IRB No. 0936-06/2024–00010556). This trial was retrospectively registered at ClinicalTrials.gov (ID: NCT07164417, Date of registration: 04/09/2025; https://clinicaltrials.gov/study/NCT07164417). All participants provided written informed consent before enrollment.

## Background

Alveolar bone is a dynamic tissue that remodels continuously in response to functional demands. Tooth extraction initiates a pronounced phase of alveolar resorption, especially within the first six months, often resulting in loss of ridge width that complicates ideal implant placement [1].

Clinically, horizontal ridge deficiency is commonly the limiting factor in placing implants in the posterior mandible [2], requiring bone augmentation procedures to restore width and allow stable implant positioning [3].

Guided bone regeneration (GBR) with particulate grafts and barrier membranes is a well-established approach to manage such defects; however, the choice of membrane and biologic adjuncts influences outcomes [4].

Autologous platelet concentrates, notably platelet-rich fibrin (PRF), have gained wide acceptance in oral and maxillofacial procedures because they are easy to prepare and deliver a spectrum of growth factors that promote angiogenesis and soft-tissue healing [5].

Conventional PRF membranes, however, are relatively rapidly resorbed (commonly within 1–2 weeks), which may limit their effectiveness as space-maintaining barrier membranes in GBR applications [6, 7].

Modified PRF preparations such as extended PRF (e-PRF) have been proposed to overcome this limitation by altering the membrane processing (thermal treatment of the plasma fraction, combined with a concentrated buffy-coat fraction) to increase matrix stability and prolong growth-factor release [8, 9].

Clinical evidence comparing PRF and e-PRF in horizontal ridge augmentation—particularly in combination with particulate xenograft and simultaneous implant placement—is limited [10].

Determining whether e-PRF provides superior volumetric and functional outcomes is clinically relevant because it could reduce reliance on synthetic or resorbable collagen barriers and improve regenerative predictability.

This randomized controlled clinical trial aimed to compare the regenerative effectiveness of conventional PRF versus e-PRF membranes used in conjunction with bovine xenograft during simultaneous implant placement for posterior mandibular horizontal ridge deficiencies. The null hypothesis was that there would be no difference in horizontal bone gain or implant stability between PRF and e-PRF groups at 6 months.

## Materials and methods

### Trial design and ethical considerations

This study was a two-arm, parallel-group randomized controlled clinical trial with a 1:1 allocation ratio. The protocol adhered to the principles of the Declaration of Helsinki and was approved by the Institutional Review Board of the Faculty of Dentistry, Alexandria University, Egypt (IRB No. 0936-06/2024–00010556). This trial was retrospectively registered at ClinicalTrials.gov (ID: NCT07164417, Date of registration: 04/09/2025; https://clinicaltrials.gov/study/NCT07164417). All participants provided written informed consent before enrollment.

The study was retrospectively registered because the research was initially designed and ethically approved as an institutional clinical investigation. Registration was completed once the importance of public trial registration for transparency and dissemination was recognized. The protocol, eligibility criteria, outcomes, and statistical analysis plan were defined before patient enrollment and were not modified after registration.

### Sample size determination

Sample size was calculated using G*Power version 3.1.9 to achieve 80% power at α = 0.05. Although horizontal bone gain was defined as the primary clinical endpoint, implant stability quotient (ISQ) was selected for a priori power calculation because no randomized clinical trials had previously reported horizontal bone gain outcomes using extended platelet-rich fibrin (e-PRF) in simultaneous implant placement with particulate xenografts, whereas validated and comparable ISQ data were available from biologically modified fibrin protocols.

Using reported mean percentage changes and standard deviations from prior studies [11, 12] (expected mean percent increase for PRF = 13.85 ± 1.75% and for an Albumin with Concentrated Growth Factor = 8.58 ± 6.42%), the pooled standard deviation was 4.08, yielding an estimated effect size of d ≈ 1.29. Based on a two-sided independent samples t-test, a minimum of 10 patients per group was required (total *N* = 20).

A post-hoc power analysis using the observed horizontal bone gain at 6 months (PRF = 0.75 ± 0.18 mm; e-PRF = 2.28 ± 0.79 mm) demonstrated that the achieved sample size provided > 90% statistical power to detect the between-group difference, indicating that the study was adequately powered for the primary outcome despite the initial ISQ-based calculation.

### Participants, settings, and eligibility criteria

Patients were recruited from the outpatient clinic of the Department of Oral and Maxillofacial Surgery, Faculty of Dentistry, Alexandria University between October 2024 and February 2025. Randomization was performed at the patient level. All implant sites within each patient received the same membrane type according to group allocation.

Inclusion criteria: adults aged 30–50 years with one or more missing posterior mandibular teeth, residual horizontal ridge width between 4 and 6 mm at the intended implant site, and dehiscence defect ≤ 5 mm.

Exclusion criteria: prior ridge reconstruction at the site, local pathology at the surgical site, medical conditions affecting healing (immunodeficiency, coagulopathies, uncontrolled diabetes), smoking > 10 cigarettes/day, pregnancy, or other contraindications to implant surgery.

### Randomization and allocation

Participants were randomly allocated into two equal groups (PRF or e-PRF) in a 1:1 ratio using a computerized random sequence generator (www.randomization.com) with variable block sizes of 4 and 6 to ensure balanced group distribution. The allocation sequence was prepared by an independent researcher not involved in participant recruitment, surgery, or outcome assessment.

Allocation concealment was ensured using sequentially numbered, opaque, sealed envelopes (SNOSE), which were prepared prior to the start of recruitment. Envelopes were opened intraoperatively only after flap reflection and confirmation of the presence of a dehiscence defect and suitability for augmentation, ensuring concealment until definitive eligibility was verified.

### Blinding

Blinding of the surgeon was not feasible because PRF and e-PRF membranes differ in preparation protocol and physical appearance. However, radiographic outcome assessors responsible for CBCT measurements and ISQ recordings were blinded to group allocation. Statistical analyses were performed using anonymized group coding, and the data analyst was blinded to treatment identity until completion of primary analyses.

Patients were not informed of the membrane type used. Postoperative care protocols and follow-up assessments were standardized to minimize expectation-related bias.

### Interventions

All patients received standardized surgical and perioperative care. Local anesthesia (articaine 4% with epinephrine 1:100,000) was administered via inferior alveolar, lingual, and long buccal nerve blocks.

**Fig. 1:**
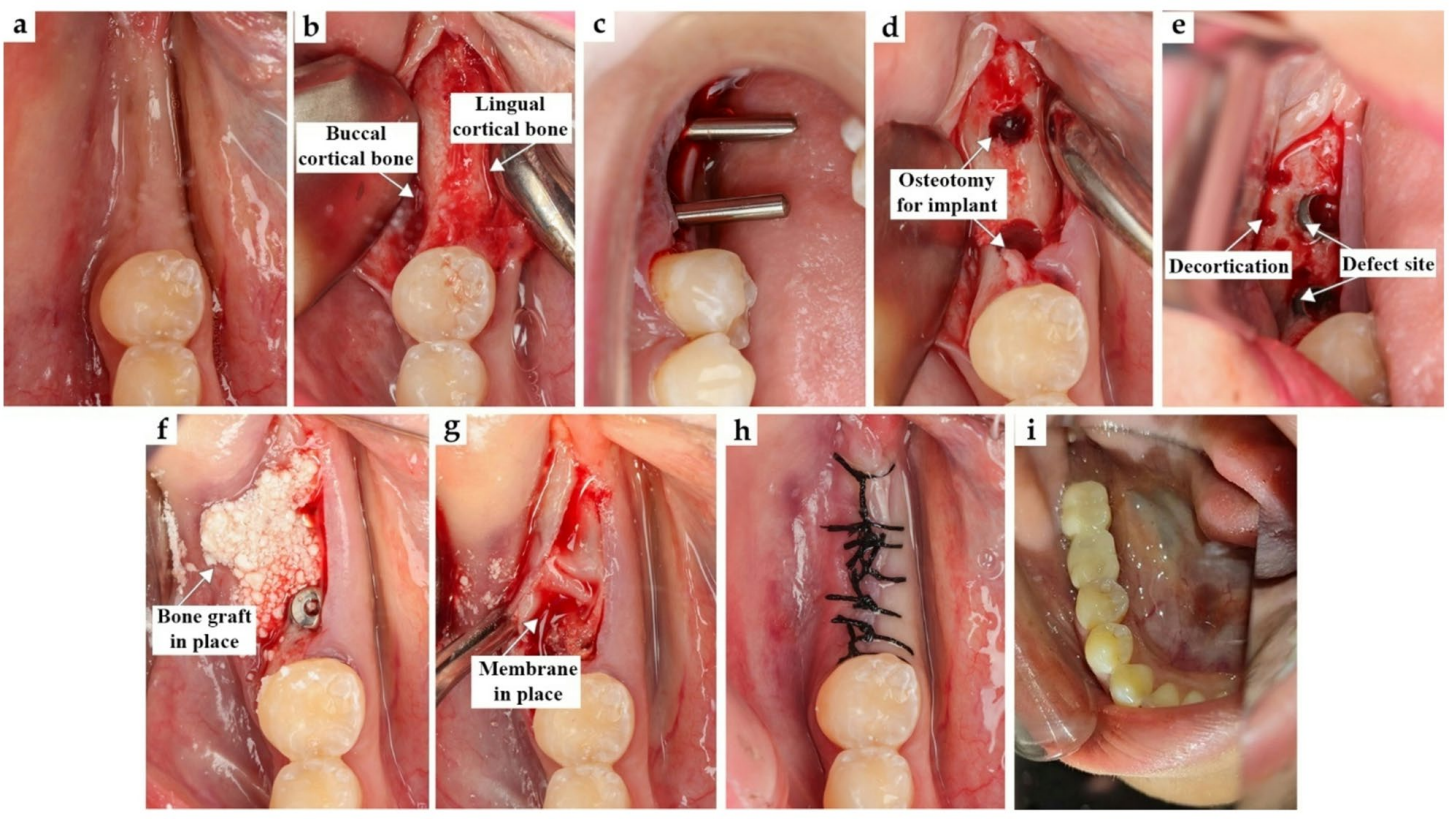
Control Group: (**a**) Preoperative clinical view, (**b**) Flap reflection, (**c**) Checking parallism, (**d**) Osteotomy, (**e**) Implant with a defect and decortication, (**f**) Bone graft on the defect, (**g**) Implant cover by PRF membrane, (**h**) Suture, (**i**) Final restoration

**Fig. 2:**
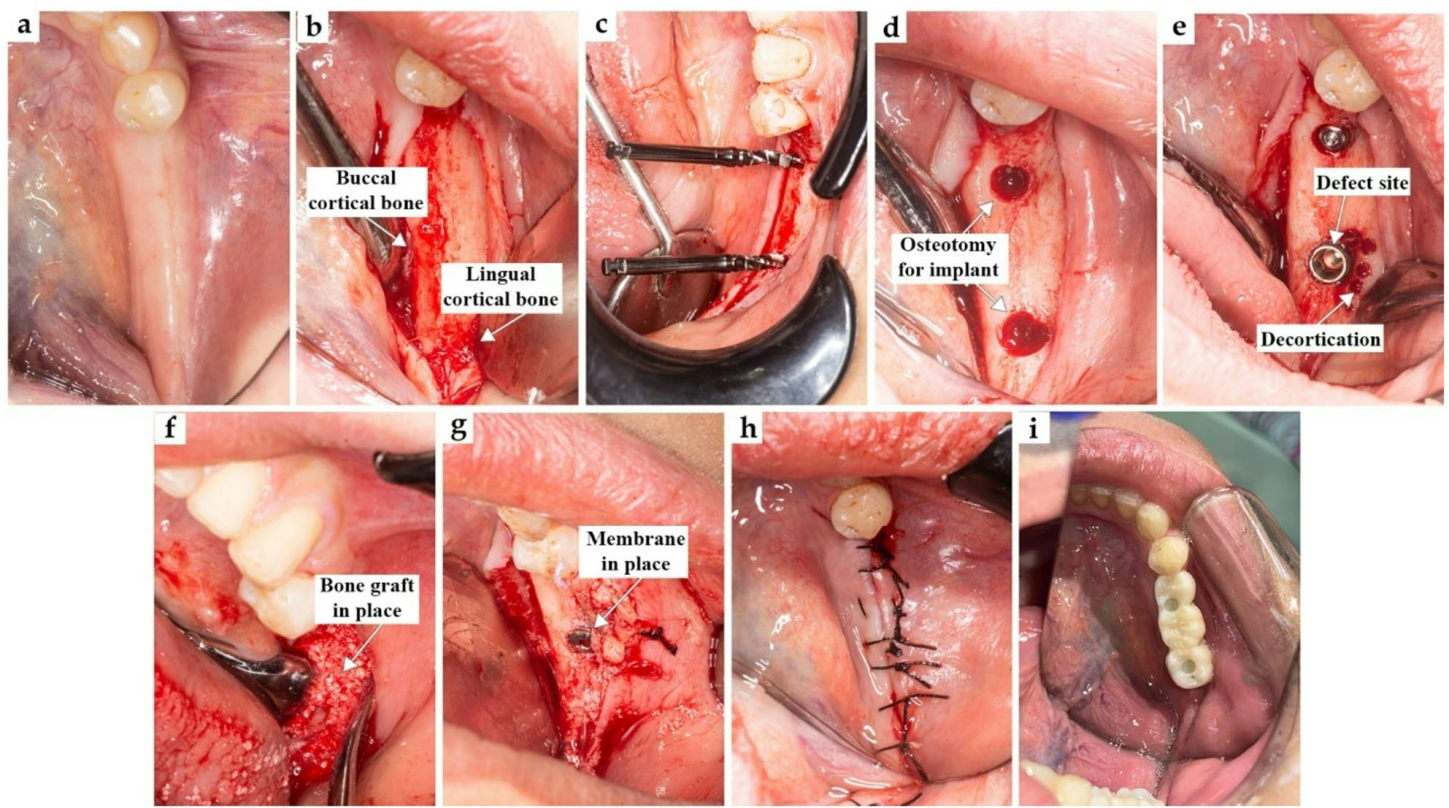
Experimental Group: (**a**) Preoperative clinical view, (**b**) Flap reflection, (**c**) Checking parallism, (**d**) Osteotomy, (**e**) Implant insertion and decortication, (**f**) Bone graft on the defect, (**g**) Implant cover by e-PRF membrane, (**h**) Suture, (**i**) Final restoration

A full-thickness triangular mucoperiosteal flap was then reflected to expose the alveolar ridge (Fig. 1a and b; Fig. 2 a and b). Sequential osteotomy was performed (Fig. 1c and d; Fig. 2c and d), and dental implants (Chaorum, Geumcheon, Korea) were inserted to the planned depth, achieving an insertion torque of up to 30 Ncm. When indicated, decortication of the buccal plate was carried out to enhance vascularization (Fig. 1e and Fig. 2e). Decortication of the buccal plate was performed when the cortical thickness was <1.5 mm or cortical sclerosis was present, as determined intraoperatively. This procedure aimed to enhance vascularization and graft integration. 

The horizontal bony defect was augmented using a particulate bovine xenograft (Onexenograft, Berlin) (Fig. 1f and Fig. 2f), followed by the application of an autologous platelet-rich fibrin–based membrane (PRF or e-PRF according to group allocation), which served as the sole barrier membrane covering the grafted site (Fig. 1g and Fig. 2g). Primary closure was achieved through periosteal releasing incisions and interrupted sutures (Fig. 1h and I; Fig. 2h and i).

For PRF membrane preparation (Control group), 9–10 mL of peripheral venous blood was collected in sterile glass tubes without anticoagulant and immediately centrifuged at 400 × g for 8 min (equivalent to approximately 2000 rpm with a rotor radius of 8 cm). The resulting fibrin clot was gently separated from the red blood cell layer and compressed using a standardized PRF compression box under constant pressure for 5 min, yielding a membrane of approximately 1 mm thickness.

In the e-PRF membrane preparation (experimental group), blood was centrifuged under the same conditions (400 × g for 8 min). The upper plasma fraction (approximately 2–4 mL) was collected and subjected to thermal treatment at 75 °C for 10 min to obtain a denatured albumin gel. After cooling to room temperature, this gel was combined with the concentrated buffy-coat fraction and allowed to homogenize for approximately 15 min. The resulting material was then compressed using the same standardized compression protocol to produce an e-PRF membrane with a target thickness of 1 mm.

Membrane thickness was visually verified using a calibrated periodontal probe before placement to ensure uniformity between patients.

In both groups, the prepared membrane was placed over the grafted area before flap repositioning and suturing. All patients received amoxicillin–clavulanic acid (1 g) twice daily for 5 days, diclofenac potassium (50 mg) three times daily for 5 days, and chlorhexidine mouthwash (0.12–0.2%) twice daily for 5 days. Cold fomentation was applied during the first 8 h postoperatively, and sutures were removed after one week.

### Outcome measures

#### Primary outcomes

**Fig. 3:**
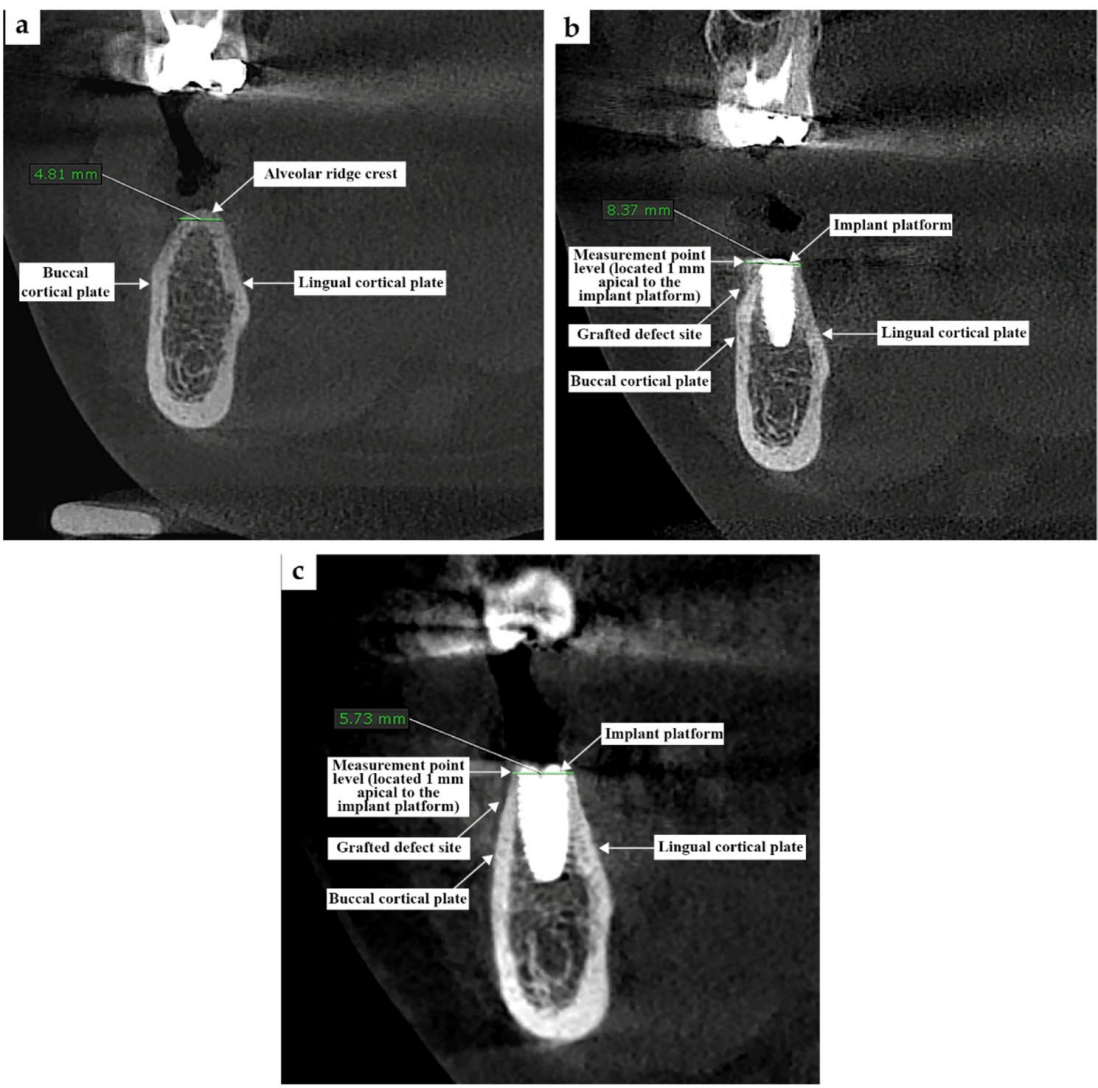
CBCT measure net: (**a**) pre-operative cone beam computed tomography (CBCT), (**b**) Immediate CBCT, (**c**) CBCT after six months

Horizontal bone width gain measured on CBCT at 6 months postoperatively. Bone width was measured at a fixed reference level: 1 mm apical to the implant platform from buccal to lingual cortical plates using OnDemand3D™ software with standardized cross-sectional reconstructions (Fig. 3a, b and c).

To assess measurement reliability, all CBCT measurements were performed by a single calibrated examiner who was blinded to group allocation. Ten randomly selected CBCT were re-measured by the same examiner after a two-week interval. Intra-examiner reliability was evaluated using the intraclass correlation coefficient (ICC), which demonstrated excellent agreement (ICC = 0.93).

#### Secondary outcomes

Implant stability (ISQ) measured by Osstell (Osstell ISQ, Stampgatan, Sweden) at baseline (primary stability recorded immediately post-insertion) and at 6 months (secondary stability), postoperative pain assessed by Visual Analogue Scale (VAS) on postoperative days 2, 3 and 7, and postoperative edema categorized clinically on the same schedule (none, intraoral local, extraoral in surgical zone, extraoral beyond surgical zone).

#### Other outcomes/complications

perioperative complications, wound dehiscence, infection, membrane exposure, or graft loss were recorded.

### Statistical analysis

Data were analyzed using IBM SPSS Statistics version 26. Normality of continuous variables was assessed using the Shapiro–Wilk test and inspection of Q–Q plots. Because randomization was performed at the patient level, all inferential statistical analyses were conducted using patient-level aggregated values. For patients who received more than one implant, the mean of implant-level measurements was calculated to obtain a single independent value per patient. This approach avoided within-patient clustering and ensured valid estimation of variance and p-values.

Bone width, implant stability quotient (ISQ), and postoperative pain values were normally distributed (*p* > 0.05) and were analyzed using parametric tests (independent and paired Student’s t-tests).

Edema scores were not normally distributed (*p* < 0.05) and were analyzed using non-parametric tests (Mann–Whitney U test, Friedman test with Dunn’s post-hoc correction). Intra-examiner reliability was evaluated using intraclass correlation coefficients (ICC). Exact p-values are reported and statistical significance was set at *p* ≤ 0.05.

## Results

**Fig. 4:**
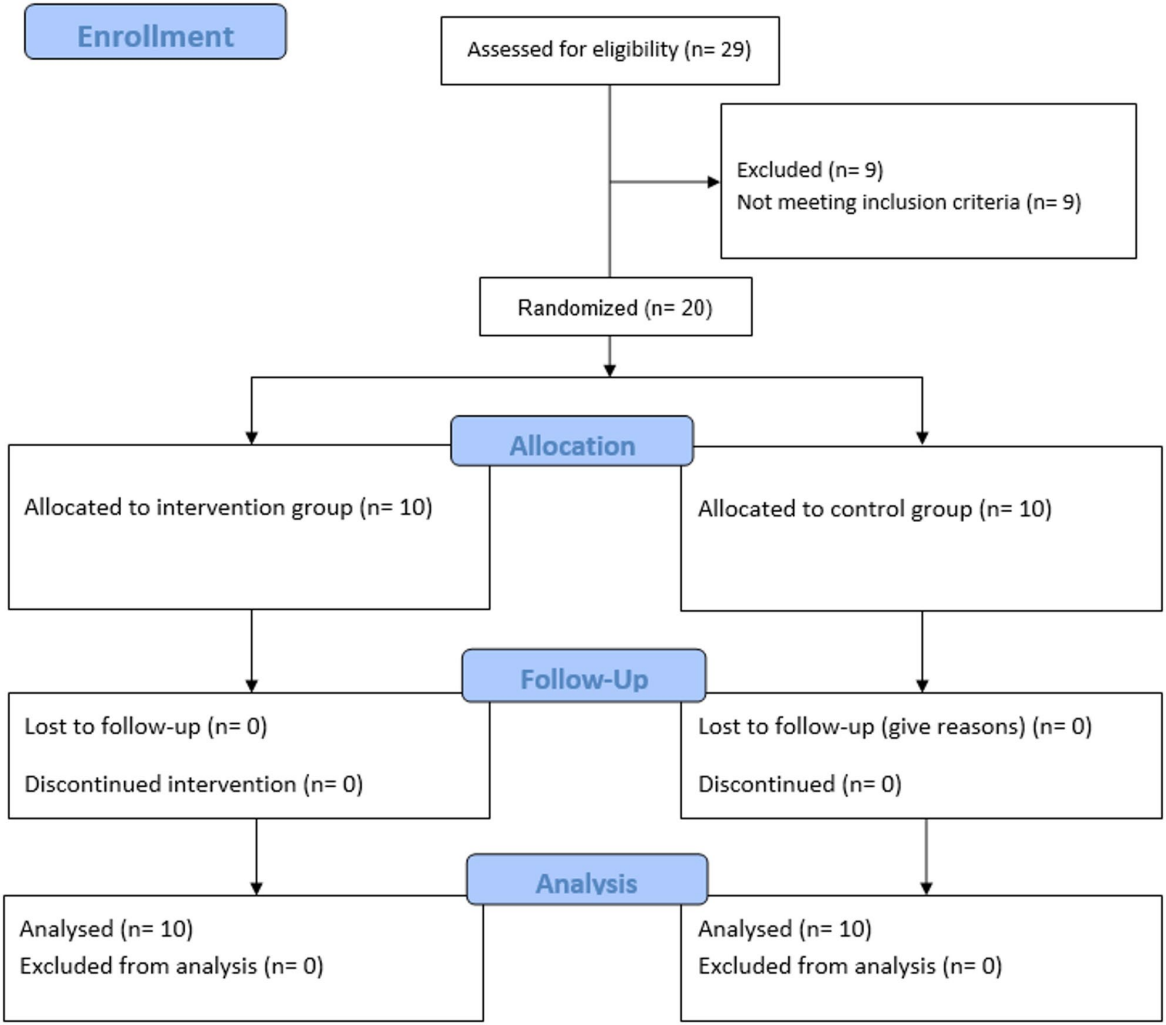
CONSORT flow diagram

Twenty patients meeting eligibility criteria were enrolled and randomized 1:1 to PRF (control, n = 10) or e-PRF (study, n = 10). All randomized participants received the assigned intervention and completed the 6-month follow-up; there were no losses to follow-up, withdrawals, or protocol deviations affecting primary outcomes (Fig. 4).

The study included 8 males and 12 females, mean age 40.2 years (range 30–49; Table 1). Thirty implants were placed (PRF group: 15 implants; e-PRF group: 15 implants. Although a total of 30 implants were placed, inferential analyses were conducted at the patient level (*n* = 20). For patients with more than one implant, mean implant values were used for statistical comparisons.

**Table 1:** Comparison between the two studied groups according to demographic data

	PRF (*n* = 10)		E PRF (*n* = 10)		Test of Sig.	*P*
	No.	%	No.	%		
Sex						
Male	5	50.0	3	30.0	ꭓ^2^=0.833	0.650
Female	5	50.0	7	70.0		
Age (years)						
Min. – Max.	32.0–49.0		30.0–49.0		t = 0.330	0.745
Mean ± SD.	41.30 ± 5.33		40.30 ± 7.94			
Median (IQR)	41.50(37.0–46.0)		41.0(33.0–48.0)			

*IQR* Inter quartile range, *SD* Standard deviation, *t* Student t-test, *χ*^2^ Chi square test

Baseline residual horizontal ridge width (mean ± SD) was 5.01 ± 0.54 mm in the PRF group and 4.98 ± 0.61 mm in the e-PRF group (no statistically significant difference, *P* = 0.914; Table 2), confirming baseline comparability.

**Table 2:** Comparison between the two studied groups according to bone width

Bone width	PRF (*n* = 10)	E PRF (*n* = 10)	T	*P* ^*^
Residual				
Min. – Max.	4.20–5.80	4.22–5.90	0.109	0.914
Mean ± SD.	5.01 ± 0.54	4.98 ± 0.61		
Median (IQR)	5.04(4.62–5.35)	4.99(4.32–5.21)		
Immediate post operative				
Min. – Max.	7.64–9.42	7.98–10.52	2.122	0.053
Mean ± SD.	8.66 ± 0.50	9.39 ± 0.95		
Median (IQR)	8.66(8.37–8.95)	9.31(8.65–10.42)		
After 6 months				
Min. – Max.	4.90–6.70	6.36–8.14	5.494^*^	0.0001^**^
Mean ± SD.	5.76 ± 0.54	7.26 ± 0.68		
Median (IQR)	5.77(5.50–5.90)	7.11(6.58–7.86)		

*IQR* Inter quartile range, *SD* Standard deviation, *t* ^*^ Independent t-test, ^**^ Statistically significant

Decortication was performed in 7 of 15 implants in the PRF group and 8 of 15 implants in the e-PRF group (*p* = 0.712, Fisher’s exact test), indicating similar distribution between groups. Given the small sample size and balanced distribution, formal subgroup or covariate-adjusted analyses were deemed unnecessary.

### Radiographic bone width

Residual (preoperative) width: PRF 5.01 ± 0.54 mm; e-PRF 4.98 ± 0.61 mm (*P* = 0.914). Immediate postoperative width: PRF 8.66 ± 0.50 mm; e-PRF 9.39 ± 0.95 mm (difference at immediate postoperative stage not clinically relevant for long-term gain; *P* = 0.053). Six months postoperative width: PRF 5.76 ± 0.54 mm; e-PRF 7.26 ± 0.68 mm (between-group difference *P* = 0.0001; Table 2).

Net horizontal bone gain (6 months vs. preop): PRF mean gain = 0.75 ± 0.18 mm; e-PRF mean gain = 2.28 ± 0.79 mm. The difference in net gain between groups was statistically significant (*P* = 0.0001; Table 2).

### Implant stability (ISQ)

Baseline (immediate postoperative) ISQ values: PRF 62.90 ± 2.60 vs. e-PRF 62.30 ± 2.75 (*P* = 0.622; Table 3). At 6 months: PRF 74.40 ± 3.06 vs. e-PRF 75.80 ± 2.30 (*P* = 0.263; Table 3). Within-group increases from baseline to 6 months were statistically significant for both groups (paired analyses, *P* = 0.0001 for each group), indicating successful osseointegration.

**Table 3:** Comparison between the two studied groups according to implant stability

Implant stability	PRF (*n* = 10)	E PRF (*n* = 10)	t	*P* ^*^
Primary				
Min. – Max.	59.0–67.0	59.0–69.0	0.501	0.622
Mean ± SD.	62.90 ± 2.60	62.30 ± 2.75		
Median (IQR)	62.50(61.0–65.0)	62.0(61.0–63.0)		
Secondary				
Min. – Max.	70.0–79.0	72.0–80.0	1.156	0.263
Mean ± SD.	74.40 ± 3.06	75.80 ± 2.30		
Median (IQR)	74.0(72.0–77.0)	75.50(74.0–77.0)		
P_1_	0.0001^*^	0.0001^*^		

*IQR* Inter quartile range, *SD* Standard deviation, ^*^Independent t-test, P_1_: p value for comparing between primary and secondary

### Postoperative pain (VAS)

VAS means decreased over time in both groups. Between-group differences were non-significant at day 2 (*P* = 0.870; Table 4), day 3 (*P* = 0.845; Table 4), and day 7 (*P* = 1.000; Table 4). Within-group repeated-measures testing showed a significant reduction across the three timepoints (*P* = 0.0001; Table 5).

**Table 4:** Comparison between the two studied groups according to pain

Pain	PRF (*n* = 10)	E PRF (*n* = 10)	T	*P* ^*^
2nd				
Min. – Max.	4.0–8.0	4.0–8.0	0.165	0.870
Mean ± SD.	5.40 ± 1.35	5.50 ± 1.35		
Median (IQR)	5.0(4.0–6.0)	5.50(4.0–6.0)		
3rd				
Min. – Max.	1.0–4.0	1.0–5.0	0.198	0.845
Mean ± SD.	2.50 ± 1.08	2.60 ± 1.17		
Median (IQR)	2.50(2.0–3.0)	2.0(2.0–3.0)		
7th				
Min. – Max.	0.0–0.0	0.0–0.0	–	1.000
Mean ± SD.	0.0 ± 0.0	0.0 ± 0.0		
Median (IQR)	0.0(0.0–0.0)	0.0(0.0–0.0)		

*IQR* Inter quartile range, *SD* Standard deviation, ^*^Independent t-test

**Table 5:** Comparison between the three studied periods according to pain

Pain	2nd	3rd	7th	F	*P* ^*^
PRF (*n* = 10)					
Min. – Max.	4.0–8.0	1.0–4.0	0.0–0.0	110.162	0.0001^**^
Mean ± SD.	5.40 ± 1.35	2.50 ± 1.08	0.0 ± 0.0		
Median (IQR)	5.0(4.0–6.0)	2.50(2.0–3.0)	0.0(0.0–0.0)		
Sig. bet. Periods^***^	P_1_=0.0001^**^, P_2_ = 0.0001^**^, P_3_ = 0.0001^**^				
E PRF (*n* = 10)					
Min. – Max.	4.0–8.0	1.0–5.0	0.0–0.0	55.390	0.0001^**^
Mean ± SD.	5.50 ± 1.35	2.60 ± 1.17	0.0 ± 0.0		
Median (IQR)	5.50(4.0–6.0)	2.0(2.0–3.0)	0.0(0.0–0.0)		
Sig. bet. periods^***^	P_1_=0.008^**^, P_2_ = 0.0001^**^, P_3_ = 0.0001^**^				

*IQR* Inter quartile range, *SD* Standard deviation, ^*^ANOVA test with repeated measures, ^**^ Statistically significant ^***^ Post Hoc Test (adjusted Bonferroni), P_1_: p value for comparing between 2nd and 3rd, P_2_: p value for comparing between 2nd and 7th, P_3_: p value for comparing between 3rd and 7th

### Postoperative edema

Edema grades postoperatively showed no significant between-group differences at days 2, 3, and 7 (*p* = 1.000; Table 6). Each group demonstrated significant within-group decreases over time (*P* = 0.0001; Table 7).

**Table 6:** Comparison between the two studied groups according to edema

Edema	PRF (*n* = 10)		E PRF (*n* = 10)		ꭓ	*P*
	No.	%	No.	%		
2nd						
None	0	0.0	0	0.0	0.00	1.000^*^
Mild	8	80.0	8	80.0		
Moderate	2	20.0	2	20.0		
Intense	0	0.0	0	0.0		
3rd						
None	0	0.0	0	0.0	1.129	1.000^**^
Mild	1	10.0	0	0.0		
Moderate	7	70.0	8	80.0		
Intense	2	20.0	2	20.0		
7th						
None	8	80.0	8	80.0	0.00	1.000^*^
Mild	2	20.0	2	20.0		
Moderate	0	0.0	0	0.0		
Intense	0	0.0	0	0.0		

^**^Fisher Exact, ^**^Monte Carlo

**Table 7:** Comparison between the two studied groups according to edema

Edema	2nd		3rd		7th		Fr	*P* ^*^
	No.	%	No.	%	No.	%		
PRF (*n* = 10)								
None	0	0.0	0	0.0	8	80.0	19.538^*^	0.0001^**^
Mild	8	80.0	1	10.0	2	20.0		
Moderate	2	20.0	7	70.0	0	0.0		
Intense	0	0.0	2	20.0	0	0.0		
Sig. bet. Periods^***^	P_1_=0.044^*^, P_2_ = 0.019^*^, P_3_ = 0.0001^*^							
E PRF (*n* = 10)								
None	0	0.0	0	0.0	8	80.0	20.0^*^	0.0001^**^
Mild	8	80.0	0	0.0	2	20.0		
Moderate	2	20.0	8	80.0	0	0.0		
Intense	0	0.0	2	20.0	0	0.0		
Sig. bet. Periods^***^	P_1_=0.025^*^, P_2_ = 0.025^*^, P_3_ = 0.0001^*^							

^*^ Friedman test, ^**^Statistically significant ^***^Post Hoc Test (Dunn’s), P_1_: p value for comparing between 2nd and 3rd, P_2_: p value for comparing between 2nd and 7th, P_3_: p value for comparing between 3rd and 7th

### Adverse events

No major adverse events, graft failures, or implant losses were reported during the 6-month follow-up. Minor postoperative events (transient pain and swelling) resolved without additional intervention.

## Discussion

This randomized controlled clinical trial enrolled 20 systemically healthy patients to compare the clinical performance of PRF and e-PRF membranes, both used in combination with xenogeneic bone grafts during simultaneous implant placement in the posterior mandible.

Implant stability, quantified by resonance frequency analysis (RFA), showed comparable mean ISQ values immediately postoperatively between the PRF and e-PRF groups. After six months, secondary stability improved significantly within both groups without a significant intergroup difference. This progressive increase in ISQ values reflects the biological transition from primary mechanical retention to secondary biological osseointegration, characterized by the formation of new bone at the implant–bone interface [13, 14]. These findings are consistent with those of Öncü and Alaaddinoğlu [15] and Farias et al. [16], confirming the reliability of ISQ as a quantitative indicator of osseointegration [17].

At baseline, ridge widths were comparable between groups, ensuring population homogeneity. Immediately after augmentation, both groups exhibited similar horizontal bone gains, indicating standardized graft placement and stable xenograft adaptation. However, after six months, a statistically significant difference emerged: the e-PRF group demonstrated a mean horizontal bone gain markedly greater than that of the PRF group. This enhanced regenerative outcome can be attributed to the prolonged bioactivity and denser fibrin network of e-PRF, which allows sustained release of growth factors such as PDGF, TGF-β, and VEGF, known to promote angiogenesis, osteoblastic differentiation, and progenitor cell recruitment [18].

The synergistic interaction between e-PRF and the xenograft likely improved graft consolidation and remodeling through enhanced cellular infiltration and neovascularization, mechanisms well documented in peri-implant regenerative healing [19].

No statistically significant intergroup differences were observed regarding postoperative pain or edema at any evaluation point. This similarity likely reflects the intrinsic biological activity of PRF and e-PRF, both enriched with autologous growth factors capable of modulating inflammation, stimulating angiogenesis, and accelerating soft tissue repair [5, 18].

Furthermore, the strict standardization of the surgical protocol—including incision design, flap management, and suturing—along with consistent perioperative care, probably minimized confounding factors influencing short-term morbidity.

Both pain and swelling peaked within the first 48–72 h and resolved completely by day 7, consistent with the findings of Babich et al. [20], who reported uneventful healing when PRF was employed in sinus floor augmentation.

When compared with the existing literature, the outcomes of this study are encouraging. Keddar et al. [21] reported horizontal bone gains of 3.2 ± 0.9 mm using particulate allografts with PRF and collagen membranes, while Park et al. [22] demonstrated greater dimensional stability when a membrane was incorporated (1.6 mm vs. 1.0 mm without membrane). Similarly, Wessing et al. [23] highlighted the impact of surgical technique, noting gains of 0.85 ± 0.35 mm without decortication and 2.98 ± 1.63 mm with decortication.

The greater horizontal bone gain observed in the e-PRF group (2.28 mm vs. 0.75 mm in the PRF group) is biologically plausible. Thermal modification of the plasma fraction and inclusion of the concentrated buffy coat result in a denser fibrin matrix, which prolongs the release of growth factors such as PDGF, TGF-β, and VEGF [8, 9, 18]. These factors stimulate angiogenesis, osteoblast differentiation, and progenitor cell recruitment, thereby supporting more effective graft consolidation [24].

Clinically, a net gain of 2 mm in horizontal ridge width is meaningful, as it can facilitate safer implant placement and improve prosthetic outcomes [21–23]. In contrast, the modest gain in the PRF group may be insufficient in more severe ridge deficiencies.

These findings support the hypothesis that thermally modified fibrin matrices can bridge the functional gap between short-lived autologous membranes and conventional collagen barriers, potentially enhancing predictability in bone augmentation procedures. Nevertheless, the relatively small sample size and limited six-month follow-up period in this study warrant caution in extrapolating long-term outcomes. Future multicenter randomized controlled trials with histomorphometry and volumetric analyses are needed to confirm the clinical consistency and long-term stability of e-PRF–assisted GBR.

A limitation of this study is that the a priori sample size calculation was based on implant stability rather than horizontal bone gain, due to the lack of published data on e-PRF at the time of study design. However, post-hoc analysis demonstrated > 90% power for the primary outcome, indicating an adequate sample size.

Additional limitations include the relatively small sample size and the 6-month follow-up period, which restrict the generalizability of the findings and preclude assessment of long-term bone stability and implant survival. The retrospective trial registration may also raise concerns regarding reporting transparency, although all outcomes were predefined and consistently applied.

Moreover, linear CBCT measurements were used instead of volumetric analysis, and the use of autologous platelet concentrates may introduce operator-dependent variability related to preparation procedures.

From a broader clinical perspective, regenerative procedures are not the sole therapeutic option for the rehabilitation of atrophic posterior mandibles. A systematic review and meta-analysis by Toti et al. demonstrated that the use of short implants can represent a clinically valid alternative to bone augmentation procedures, offering comparable survival rates with reduced surgical morbidity [25]. Similarly, Vinci et al. highlighted that inferior alveolar nerve repositioning, digitally guided implant placement, piezoelectric surgical approaches, and immediate prosthetic protocols constitute established alternatives that may circumvent the need for regenerative augmentation in selected cases [26]. 

Within this therapeutic spectrum, e-PRF–assisted guided bone regeneration should be regarded as a biologically driven option particularly suited for patients in whom conventional implant placement is limited by horizontal ridge deficiency but where nerve repositioning or the use of short implants is not ideal due to prosthetic or biomechanical considerations. The enhanced horizontal bone gain observed in the present trial suggests that e-PRF may improve the predictability of minimally invasive regenerative protocols while preserving the advantages of autologous, biologically active membranes.

Future multicenter randomized clinical trials with larger sample sizes, longer follow-up periods, volumetric CBCT or histomorphometric analyses, and standardized PRF preparation protocols are required to further define the long-term stability, clinical indications, and cost-effectiveness of e-PRF-assisted regeneration in posterior mandibular rehabilitation.

In summary, the present trial provides early clinical evidence that e-PRF membranes, by virtue of their enhanced mechanical integrity and sustained growth factor release, can significantly improve horizontal bone regeneration when combined with xenogeneic grafts and immediate implant placement. While both PRF and e-PRF yielded comparable outcomes in terms of early healing and implant stability, e-PRF demonstrated more predictable bone gain, positioning it as a promising next-generation biomaterial for biologically driven ridge augmentation.

## Conclusion

Both PRF and e-PRF membranes, when used with bovine xenograft and simultaneous implant placement, supported favorable clinical healing and progressive implant stability. Extended PRF achieved significantly greater horizontal bone gain at 6 months, reflecting its potential as a biologically active adjunct for horizontal ridge augmentation. No differences were observed between groups in implant stability, postoperative pain, edema, or minor complications. These findings are limited to short-term radiographic outcomes, and further long-term, volumetric, and clinically oriented studies are required to confirm sustained bone stability and clinical effectiveness.

## Data Availability

The data provided for the results presented in this study is available through the corresponding author upon request.

## Abbreviations

PRF: Platelet-Rich Fibrin
e-PRF: Extended Platelet-Rich Fibrin
GBR: Guided Bone Regeneration
CBCT: Cone-Beam Computed Tomography
ISQ: Implant Stability Quotient
VAS: Visual Analogue Scale
SD: Standard Deviation
IQR: Interquartile Range
SPSS: Statistical package for social sciences
IRB: Institutional Review Board
RFA: Resonance Frequency Analysis
PDGF: Platelet-Derived Growth Factor
TGF-β: Transforming Growth Factor-Beta
VEGF: Vascular Endothelial Growth Factor
